# Real-world evidence on spinal cord neuromodulation and pain: Long-term effectiveness analysis in a single-center cohort

**DOI:** 10.1016/j.bas.2021.100301

**Published:** 2021-10-23

**Authors:** José Manuel Viñuela-Prieto, José Francisco Paz-Solís, Alberto Isla-Guerrero, Javier Díaz-de-Terán, María Luisa Gandía-González

**Affiliations:** a Neurosurgery Department, Hospital Universitario La Paz, Madrid, Spain; b Neurology Department, Hospital Universitario La Paz, Madrid, Spain; c CranioSPain Research Group, Centro Superior de Estudios Universitarios La Salle, Madrid, Spain; d Hospital La Paz Institute for Health Research, IdiPaz, Madrid, Spain

**Keywords:** Spinal cord stimulation, Spinal chronic pain, Neuromodulation, Real-world evidence

## Abstract

**Introduction:**

Chronic pain inflicts damage in multiple spheres of patient's life and remains a challenge for health care providers. Real-world evidence derived from outcome registries represents a key aspect of the ongoing systematic assessment and future development of neurostimulation devices.

**Research question:**

The objective of the present study was to assess the long-term effectiveness of neurostimulation as a treatment for spinal chronic pain.

**Material and methods:**

The patients analyzed in the present study represent a singlecenter cohort of 52 individuals. Primary outcome measures included numeric pain rating scale, Beck depression index II and Oswestry disability index variation from baseline to 36-month visits. Secondary outcomes included its evaluation at 6-month, 12-month and 24-month visits.

**Results:**

A significant improvement in targeted pain, depression and disability values were observed at 36-month follow-up (P ​< ​0.001, P ​= ​0.009 and P ​< ​0.001 respectively). Those results were consistent in the leg and back pain subgroup but not in the neck, chest and arm pain subgroup. The decrease in pain, depression and disability values happened progressively through time, with the exception of the 12-month visit, where a mild stagnation was observed.

**Discussion and conclusion:**

Our results suggest that spinal cord stimulation is an effective long-term treatment for spinal chronic pain in real-world conditions when applied to a variety of patients and conditions usually seen in routine practice. Nevertheless, some fluctuations may occur during treatment so prolonged follow-up periods should be considered before rendering an unsuccessful therapy diagnosis.

## Highlights

• Spinal cord stimulation is a treatment option for spinal chronic pain.
• Real-world evidence from large registries regarding its effectiveness is needed.
• Long-term improvement in pain, depression and disability can be achieved.
• Fluctuations during treatment may occur, but do not limit the potential benefit.

## 1 Introduction

The prevalence of chronic spinal pain has significantly increased over recent years owing to population aging, increasingly sedentary lifestyle habits, obesity and smoking, among others (Kao et al., 2021; ​Manchikanti et al., 2021; Manchikanti et al., 2014). Specifically, low back and neck pain, associated or not to lower and upper extremity pain respectively, affects nearly a 20% of the population and are frequently associated with depression, headache and osteoporosis (Manchikanti et al., 2014; Fernández-de-las-Peñas et al., 2011; Yong et al., 2021).

A myriad of treatment modalities for chronic pain have been developed over the centuries, including both pharmacological and non-pharmacological approaches (Cohen et al., 2021). In that sense, the effectiveness and cost-benefit ratio of the available treatment options raise great concern to public and private healthcare systems worldwide (Hylands-White et al., 2017).

Among those therapeutic options, neurostimulation therapy has been classically reserved for those patients with intractable pain that have not responded to pharmaco-logical and/or other conservative therapies (Provenzano et al., 2021; Knotkova et al., 2021). Spinal cord stimulation (SCS) applies electric currents in the epidural space surrounding the dorsal columns of the spinal cord. Those electric pulses generate a paresthesia feeling along the dermatome corresponding to the targeted spinal cord level. Its efficacy has been already demonstrated for patients suffering from failed back surgery syndrome (FBSS) (Kumar et al., 2007), complex regional pain syn-drome (Knotkova et al., 2021) and peripheral neuropathies with radicular symptoms (Provenzano et al., 2021), among other types of pain. The mechanism of action of SCS has been classically explained by the Gate Control Theory developed by Melzack and Wall back in 1965 (Melzack and Wall, 1965). However, the appearance of modalities that do not depend on paresthesia has given rise to alternative explanations of the mechanism of action of the SCS (e. g. supraspinal pathways activation and segmental modulation) (Vallejo et al., 2020).

When recommending SCS as a treatment option for chronic pain, some authors express their reservations regarding the limited data on effectiveness beyond 12 months of follow-up, where some tapering of the effects have been reported (Knotkova et al., 2021).

The objective of the present study was to assess the long-term (up to 3 years) effectiveness of SCS as a treatment for chronic pain in routine clinical practice at a high-volume Spanish hospital.

## 2 Materials and methods

### 2.1 Study design

The present was a prospective, single-center study meant to collect the characteristics of the actual clinical outcomes of Boston Scientific's (Boston Scientific Neuromodulation, CA, USA) commercially licensed neurostimulation systems for pain in routine clinical practice. The study retrieved information from patients that received a permanent SCS implant for pain control. The approval from our center's ethics committee (EC) and written informed consent from every patient included in the study, delivered in Spanish, were obtained.

### 2.2 Inclusion and exclusion criteria

Key inclusion criteria included adults (age 18 or older when written informed consent is obtained), who are scheduled to be trialed, on-label, with a commercially approved neurostimulation system for pain and who are willing and able to comply with the study requirements and sign a valid, EC-approved informed consent form.

Key exclusion criteria included patients with any contraindication for SCS system implantation or currently diagnosed with cognitive impairment, or exhibiting any characteristic, that would limit study candidate's ability to assess pain relief or to complete study assessments.

### 2.3 Implantation, follow-up and study duration

Before implantation, patients underwent a psychological assessment carried out by a professional psychologist where the ability to assume the management of the device, the presence of severe psychological disorders as well as unresolved issues of secondary gain were evaluated. The SCS electrodes and stimulators were implanted following the standard technique according to the product manuals. A trial period with placement of the SCS leads powered by an external neurostimulator prior to implantable pulse generator (IPG) activation was performed. Patients whose neurostimulation test was unsuccessful -defined as either pain reduction from baseline of less than 3 numeric pain rating scale (NPRS) points or describing paresthesia as uncomfortable and disagreeing to proceed to permanent implant- or who have not undergone IPG implantation at 12 months after the neurostimulation trial visit will terminate their participation in the registry.

Follow-up evaluations were carried out at baseline, 6-, 12-, 24- and 36-months visits.

### 2.4 Clinical outcomes

The primary outcome was change from baseline in pain intensity, Beck depression inventory-second edition (BDI-II) (Beck et al., 1996) and Oswestry disability index version 2.1a (ODI v2.1a) (Fairbank and Pynsent, 2000; Fairbank, 2014) scores at 36 months. The secondary outcome was the change in those same variables from baseline to 6-, 12- and 24-months visits.

Pain intensity was measured by the NPRS, an 11-point scale, where 0 indicates no pain and 10 indicates the worst pain imaginable. The BDI-II includes 21 items valued on a 4-point Likert scale ranged from 0 to 3, according to the intensity in the preceding two weeks. Total score ranged from 0 to 63, with higher scores indicating greater symptoms’ severity (Beck et al., 1996). The ODI v2.1a comprises 10 items and is one of the most common patient-reported outcome measures used to assess disturbance caused by chronic pain (Fairbank and Pynsent, 2000; Fairbank, 2014).

Additional outcomes measures were assessed by means of the paresthesia coverage percentage, the development of new areas of neurostimulation-amenable pain, the patient global impression of change (PGIC), clinician global impression of change (CGIC) and subject satisfaction with stimulation treatment (SST) at all follow up time points.

Variables regarding basic demographic, medical history, physical exam and implantation/activation procedures were also collected in order to better define our cohort's characteristics. Adverse events and device components deficiencies were also monitored throughout the study.

No data imputation was performed if the patient missed any visit.

### 2.5 Statistical analysis

Baseline characteristics were presented using appropriate descriptive statistics.

Continuous parametric data were presented as mean ​± ​SD and analyzed using the paired Student *t*-test or the repeated measures analysis of variance (ANOVA) with post-hoc Bonferroni corrections for multiple comparisons. Mauchly's sphericity test was used for testing sphericity and the Greenhouse-Geisser correction was used in the case of lack of sphericity. For continuous non-parametric data, Wilcoxon signed-rank test was employed. Cochran's Q with post-hoc corrections for multiple comparisons was used to compare differences between more than two proportions. Non-parametric ordinal data was assessed with Spearman's (rho) rank-order test.

Linear regression model was built to evaluate the influence of independent variables such as age, gender, time from pain onset to implantation, previous spine surgeries, medication, diabetes, smoking, BMI, targeted pain, SF-MPQ, BDI-II and ODI values at baseline on the difference in pain intensity measured with NPRS from baseline to 36-month visit (dependent variable). Durbin-Watson test was used to detect autocorrelation in the residuals. In addition, the presence of collinearity was discarded using the variance inflation factor (VIF). To find evidence of heteroskedasticity we employed the Breusch-Pagan test.

The level of significance was defined as P ​< ​0.05. All statistical analysis were performed using SPSS® Version 20 (IBM®, Chicago, USA).

## 3 Results

**Fig. 1:**
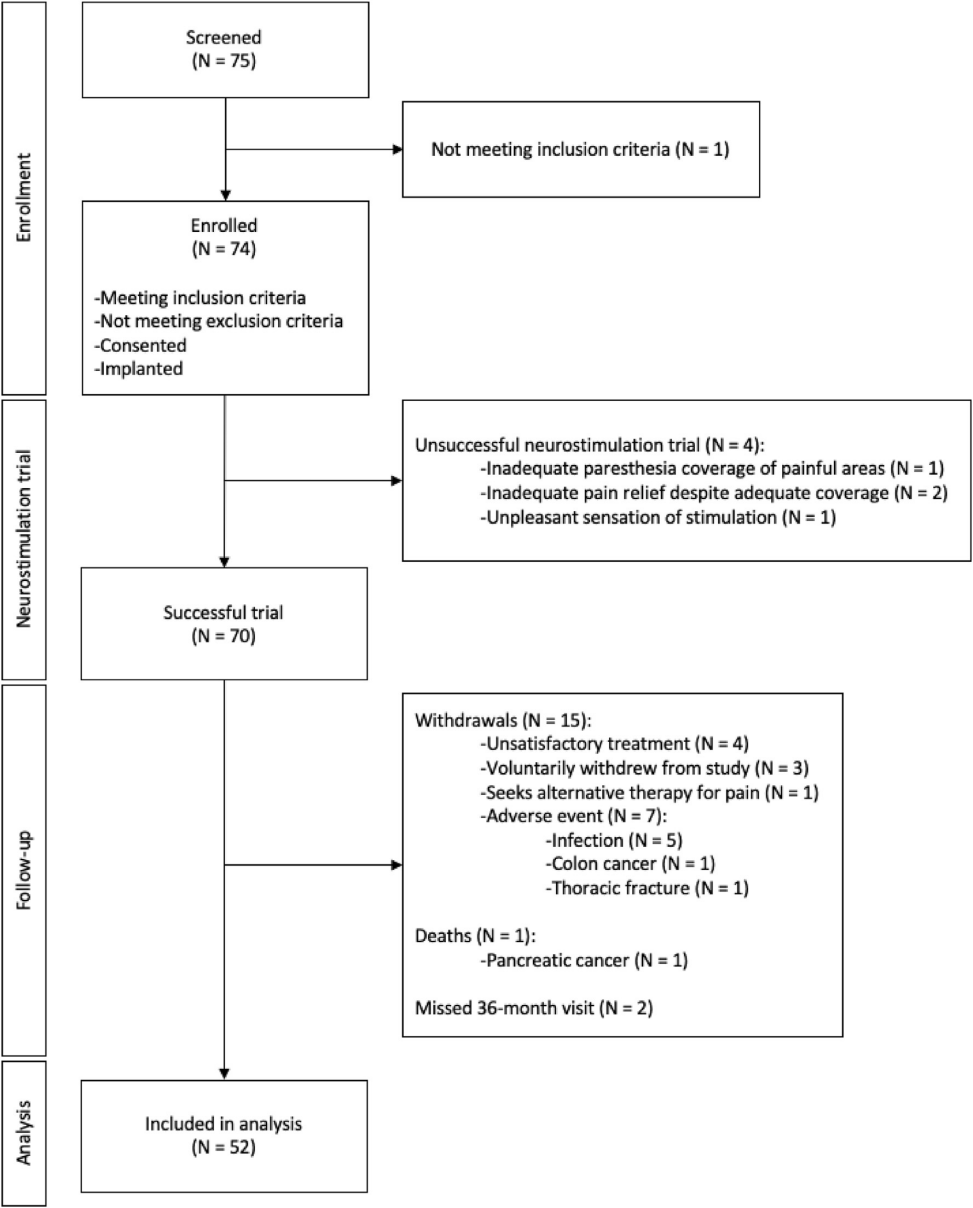
Flow diagram of cohort generation.

Between November 2013 and April 2016, a total of 75 patients were screened for their inclusion in the study. Of them, 74 were implanted and subjected to a neurostimulation trial that resulted successful in 70 cases. The cohort used in the analysis was constructed after subtracting the patients withdrawn from the study, dead during follow-up and those that missed the 36-month visit. The flow diagram of the cohort generation is depicted in Fig. 1.

### 3.1 Cohort characteristics

Results regarding demographic, medical history, physical exam and implantation/activation procedures' data are summarized in Table 1.

**Table 1:** Demographics, medical history, physical exam and implantation/activation procedures' characteristics.

Demographics	
Age at time of consent (years; mean ​± ​SD)	52.35 ​± ​13.96
**Gender male [N (%)]**	27 (51.9)
**Ethnicity [N (%)]**	
Caucasian	49 (94.2)
Hispanic or Latino	3 (5.8)
**Medical history**	
**Time from pain onset to implantation (years; mean ​± ​SD)**	6.92 ​± ​5.70
**Time from pain onset to implantation [years; median (IQR)]**	5 (3.25–8)
**Patients with previous spine surgeries [N (%)]**	41 (78.8)
**Number of previous spine surgeries per patient [median (IQR)]**	2 (1–2)
**Indication for neurostimulation [N (%)]**	
Low back and/or unilateral or bilateral lower extremity pain:	43 (82.7)
Isolated low back pain	2 (3.8)
Isolated unilateral lower extremity pain	5 (9.6)
Isolated bilateral lower extremity pain	4 (7.7)
Low back ​+ ​unilateral lower extremity pain	13 (25)
Low back ​+ ​bilateral lower extremity pain	19 (36.5)
	
Neck and/or unilateral or bilateral upper extremity pain:	9 (17.3)
Isolated unilateral upper extremity pain	7 (13.5)
Neck ​+ ​bilateral upper extremity pain	2 (3.8)
**SF-MPQ 2 (mean ​± ​SD)**	99.65 ​± ​38.26
**Pain related diagnosis - Central neuropathic pain [N (%)]**	
No	50 (96.2)
Compressive myelopathy from spinal stenosis	1 (1.9)
Post-traumatic spinal cord injury pain	1 (1.9)
**Pain related diagnosis - Peripheral neuropathic pain [N (%)]**	
No	4 (7.7)
Failed Back Surgery Syndrome	25 (48.1)
Lumbosacral radiculopathy	1 (1.9)
Failed Back Surgery Syndrome ​+ ​Lumbosacral radiculopathy	11 (21.2)
Cervical radiculopathy	1 (1.9)
Complex Regional Pain Syndrome	7 (13.5)
Post-traumatic neuralgias	2 (3.8)
Other	1 (1.9)
**Pain related diagnosis - Somatic pain [N (%)]**	
No	51 (98.1)
Peripheral vascular diseases	1 (1.9)
**Passed psychological evaluation [N (%)]**	41 (78.8)
**Untreated anxiety disorder [N (%)]**	0 (0)
**Somatoform anxiety disorder [N (%)]**	0 (0)
**Diabetes mellitus [N (%)]**	4 (7.7)
**Current illicit drug/alcohol abuse [N (%)]**	0 (0)
**Smoker [N (%)]**	
Never	28 (53.8)
Previous	7 (13.5)
Current	16 (30.8)
**Unresolved issues of secondary gain, e.g. litigation [N (%)]**	0 (0)
**Past invasive procedure for chronic pain condition [N (%)]**	47 (90.4)
**Past non-invasive treatment for chronic pain condition [N (%)]**	48 (92.3)
**Medication at baseline (N; mean ​± ​SD)**	2.79 ​± ​1.58
Non-opioid analgesics [N (%)]	24 (46.2)
NSAIDs [N (%)]	10 (19.2)
Opioids [N (%)]	35 (67.3)
Anticonvulsants [N (%)]	27 (51.9)
Antidepressants [N (%)]	19 (36.5)
Muscle relaxants [N (%)]	15 (28.8)
**Medication ceased during SCS treatment [N (%)]**	
None	45 (86.5)
1	2 (3.8)
2	1 (1.9)
3 or more	1 (1.9)
**Physical exam**	
**Height (cm; mean ​± ​SD)**	166.67 ​± ​9.23
**Weight (kg; mean ​± ​SD)**	76.00 ​± ​15.28
**BMI (kg/m**^**2**^**; mean ​± ​SD)**	27.32 ​± ​4.79
**Anatomical abnormality near surgical implant site [N (%)]**	0 (0)
**Spinal abnormalities [N (%)]**	1 (1.9)
**Coagulation abnormality [N (%)]**	0 (0)
**Evidence of thoracolumbar myelopathy [N (%)]**	0 (0)
**Evidence of lumbosacral radiculopathy [N (%)]**	36 (69.2)
**Evidence of peripheral neuropathy [N (%)]**	12 (23.1)
**Implantation/Activation procedures**	
**Time from implantation to final configuration (days; mean ​± ​SD)**	9.40 ​± ​5.49
**Time from implantation to final configuration [days; median (IQR)]**	8.50 (6–14)
**Device components - Lead [N (%)]**	
Cover Edge™ 1x32	2 (3.8)
Infinion™ 1x16	35 (67.3)
Linear ST™ 1x8	15 (28.9)
**Lead location [N (%)]**	
C4	3 (5.8)
C5	1 (1.9)
C6	2 (3.8)
C7	1 (1.9)
C8	1 (1.9)
T1	1 (1.9)
T5	1 (1.9)
T7	17 (32.7)
T8	18 (34.6)
T9	4 (7.7)
T10	1 (1.9)
T11	1 (1.9)
**Device components - IPG [N (%)]**	
Precision™ Plus	28 (53.8)
Precision Spectra™	24 (46.2)
**Total [N (%)]**	52 (100)

Abbreviations: SD: standard deviation; IQR: interquartile range; SF-MPQ 2: short-form McGill pain questionnaire 2; IPG: implantable pulse generator.

### 3.2 Primary outcome

When the whole cohort of patients was considered, targeted pain intensity, BDI-II and ODI scores significantly decreased from baseline to 36-month visit after implantation (P ​< ​0.001, P ​= ​0.009 and P ​< ​0.001 respectively).

**Fig. 2:**
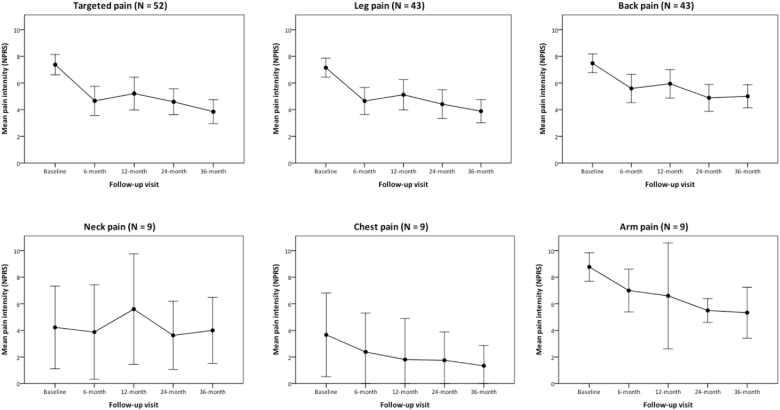
Evolution in mean pain intensity over time by pain location. NPRS: numeric pain rating scale. Error bars represent 95% CI of the mean.

In the subgroup of patients in whom the indication for neurostimulation was either back and/or lower extremity pain, back and leg pain intensity along with BDI-II and ODI scores also decreased significantly (P ​< ​0.001, P ​< ​0.001, P ​= ​0.012 and P ​< ​0.001 respectively). Nevertheless, in the subgroup of patients suffering from neck and/or upper extremity pain, only the arm pain intensity showed a statistically significant reduction from baseline to the 36-month follow-up visit (P ​= ​0.020) (Fig. 2).

Results regarding primary outcome measures are summarized in Table 2.

**Table 2:** Primary outcome measures: change in pain intensity, BDI-II and ODI scores from baseline to 36-month visit.

Variable^a^	N	Baseline	36-month visit	Mean difference (95% CI)	Statistic^b^	P-value
All patients (N ​= ​52)						
**Targeted pain**	41	7.41 ​± ​2.68	3.85 ​± ​3.01	3.56 (2.23–4.90)	5.389	<0.001^c^
**BDI-II**	52	19.40 ​± ​11.06	15.17 ​± ​12.62	4.23 (1.12–7.34)	2.728	0.009^c^
**ODI**	45	50.84 ​± ​14.09	34.93 ​± ​19.65	15.91 (9.86–21.97)	5.296	<0.001^c^
**Back and/or unilateral or bilateral lower extremity pain (N = 43)**						
**Leg pain**	41	7.15 ​± ​2.27	3.88 ​± ​2.90	3.27 (2.42–4.12)	7.772	<0.001^c^
**Back pain**	41	7.61 ​± ​2.10	5.12 ​± ​2.70	2.49 (1.46–3.51)	4.903	<0.001^c^
**BDI-II**	43	18.51 ​± ​10.83	14.16 ​± ​11.17	4.35 (1.01–7.69)	2.625	0.012^c^
**ODI**	42	50.52 ​± ​13.44	33.52 ​± ​18.96	17.00 (10.65–23.36)	5.403	<0.001^c^
**Neck and/or unilateral or bilateral upper extremity pain (N = 9)**						
**Neck pain**	9	4.22 ​± ​4.06	4.00 ​± ​3.24	-	−0.271	0.786^d^
**Chest pain**	9	3.67 ​± ​4.09	1.33 ​± ​2.00	-	−1.472	0.141^d^
**Arm pain**	9	8.78 ​± ​1.39	5.33 ​± ​2.50	-	−2.320	0.020^d^
**BDI-II**	9	23.67 ​± ​11.81	20.00 ​± ​18.16	-	−0.771	0.440^d^
**ODI**	9	40.89 ​± ​20.23	54.67 ​± ​22.30	-	−0.272	0.785^d^

Abbreviations: CI: confidence interval; BDI-II: Beck depression inventory – second edition; ODI: Oswestry disability index version 2.1a.

a Expressed as mean ​± ​SD.

b T-statistic for paired Student *t*-test and Z-statistic for Wilcoxon signed rank test**.**

c Paired Student *t*-test.

d Wilcoxon signed rank test.

### 3.3 Secondary outcomes

Table 3, Table 4, Table 5 summarize the results regarding secondary outcome measures at 6, 12 and 24-month follow-up visits.

**Table 3:** Secondary outcome measures: change in pain intensity and ODI scores from baseline to 6-month visit.

Variable^a^	N	Baseline	6-month visit	Mean difference (95% CI)	Statistic^b^	P-value
All patients (N ​= ​52)						
**Targeted pain**	32	6.94 ​± ​2.85	4.69 ​± ​3.15	2.25 (0.77–3.73)	3.102	0.004^c^
**ODI**	49	49.02 ​± ​14.91	40.24 ​± ​18.13	8.78 (4.07–13.48)	3.751	<0.001^c^
**Back and/or unilateral or bilateral lower extremity pain (N = 43)**						
**Leg pain**	34	6.97 ​± ​2.37	4.65 ​± ​2.90	2.32 (1.54–3.11)	6.015	<0.001^c^
**Back pain**	34	7.41 ​± ​2.43	5.59 ​± ​3.05	1.82 (0.90–2.75)	4.019	<0.001^c^
**ODI**	41	50.24 ​± ​13.48	40.78 ​± ​17.22	9.46 (3.93–15.00)	3.456	0.001^c^
**Neck and/or unilateral or bilateral upper extremity pain (N = 9)**						
**Neck pain**	9	4.22 ​± ​4.06	3.88 ​± ​4.26	-	−0.271	0.786^d^
**Chest pain**	9	3.67 ​± ​4.09	2.38 ​± ​3.50	-	−0.944	0.345^d^
**Arm pain**	9	8.78 ​± ​1.39	7.00 ​± ​1.93	-	−1.930	0.054^d^
**ODI**	9	40.89 ​± ​20.23	37.50 ​± ​23.44	-	−1.546	0.122^d^

Abbreviations: CI: confidence interval; ODI: Oswestry disability index version 2.1a.

a Expressed as mean ​± ​SD.

b T-statistic for paired Student *t*-test and Z-statistic for Wilcoxon signed rank test**.**

c Paired Student *t*-test.

d Wilcoxon signed rank test.

**Table 4:** Secondary outcome measures: change in pain intensity, BDI-II and ODI scores from baseline to 12-month visit.

Variable^a^	N	Baseline	12-month visit	Mean difference (95% CI)	Statistic^b^	P-value
All patients (N ​= ​52)						
**Targeted pain**	29	6.97 ​± ​3.10	5.52 ​± ​3.40	1.45 (0.01–2.89)	2.060	0.049^c^
**BDI-II**	49	18.55 ​± ​10.52	17.24 ​± ​13.05	1.31 (−2.38–4.99)	0.713	0.479^c^
**ODI**	47	48.30 ​± ​15.46	39.66 ​± ​20.39	8.64 (3.23–14.05)	3.213	0.002^c^
**Back and/or unilateral or bilateral lower extremity pain (N = 43)**						
**Leg pain**	32	7.00 ​± ​2.40	5.16 ​± ​3.31	1.84 (0.85–2.84)	3.778	0.001^c^
**Back pain**	33	7.24 ​± ​2.44	6.12 ​± ​2.95	1.12 (0.20–2.04)	2.482	0.019^c^
**BDI-II**	40	17.40 ​± ​10.02	17.15 ​± ​12.92	0.25 (−4.03–4.53)	0.118	0.907^c^
**ODI**	38	50.05 ​± ​13.87	41.42 ​± ​19.46	8.63 (2.03–15.23)	2.650	0.012^c^
**Neck and/or unilateral or bilateral upper extremity pain (N = 9)**						
**Neck pain**	9	4.22 ​± ​4.06	5.60 ​± ​3.36	-	−0.378	0.705^d^
**Chest pain**	9	3.67 ​± ​4.09	1.80 ​± ​2.49	-	−0.365	0.715^d^
**Arm pain**	9	8.78 ​± ​1.39	6.60 ​± ​3.21	-	−1.095	0.273^d^
**BDI-II**	9	23.67 ​± ​11.81	17.67 ​± ​14.42	-	−1.721	0.085^d^
**ODI**	9	40.89 ​± ​20.23	32.22 ​± ​23.70	-	−2.077	0.038^d^

Abbreviations: CI: confidence interval; BDI-II: Beck depression inventory – second edition; ODI: Oswestry disability index version 2.1a.

a Expressed as mean ​± ​SD.

b T-statistic for paired Student *t*-test and Z-statistic for Wilcoxon signed rank test.

c Paired Student *t*-test.

d Wilcoxon signed rank test.

**Table 5:** Secondary outcome measures: change in pain intensity and ODI scores from baseline to 24-month visit.

Variable^a^	N	Baseline	24-month visit	Mean difference (95% CI)	Statistic^b^	P-value
All patients (N ​= ​52)						
**Targeted pain**	34	7.15 ​± ​2.91	4.74 ​± ​2.87	2.41 (1.19–3.64)	3.998	<0.001^c^
**ODI**	49	48.45 ​± ​15.26	32.27 ​± ​18.54	15.88 (10.34–21.42)	5.762	<0.001^c^
**Back and/or unilateral or bilateral lower extremity pain (N = 43)**						
**Leg pain**	32	7.13 ​± ​2.51	4.41 ​± ​3.14	2.72 (1.67–3.76)	5.306	<0.001^c^
**Back pain**	33	7.48 ​± ​2.09	5.03 ​± ​2.81	2.46 (1.49–3.42)	5.168	<0.001^c^
**ODI**	41	50.29 ​± ​13.52	33.12 ​± ​17.26	17.17 (10.69–23.65)	5.356	<0.001^c^
**Neck and/or unilateral or bilateral upper extremity pain (N = 9)**						
**Neck pain**	9	4.22 ​± ​4.06	3.63 ​± ​3.07	-	−0.674	0.500^d^
**Chest pain**	9	3.67 ​± ​4.09	1.75 ​± ​2.55	-	−1.214	0.225^d^
**Arm pain**	9	8.78 ​± ​1.39	5.50 ​± ​1.07	-	−2.533	0.011^d^
**ODI**	9	40.89 ​± ​20.23	29.75 ​± ​25.38	-	−1.902	0.057^d^

Abbreviations: CI: confidence interval; ODI: Oswestry disability index version 2.1a.

a Expressed as mean ​± ​SD.

b T-statistic for paired Student *t*-test and Z-statistic for Wilcoxon signed rank test**.**

c Paired Student *t*-test.

d Wilcoxon signed rank test.

**Fig. 3:**
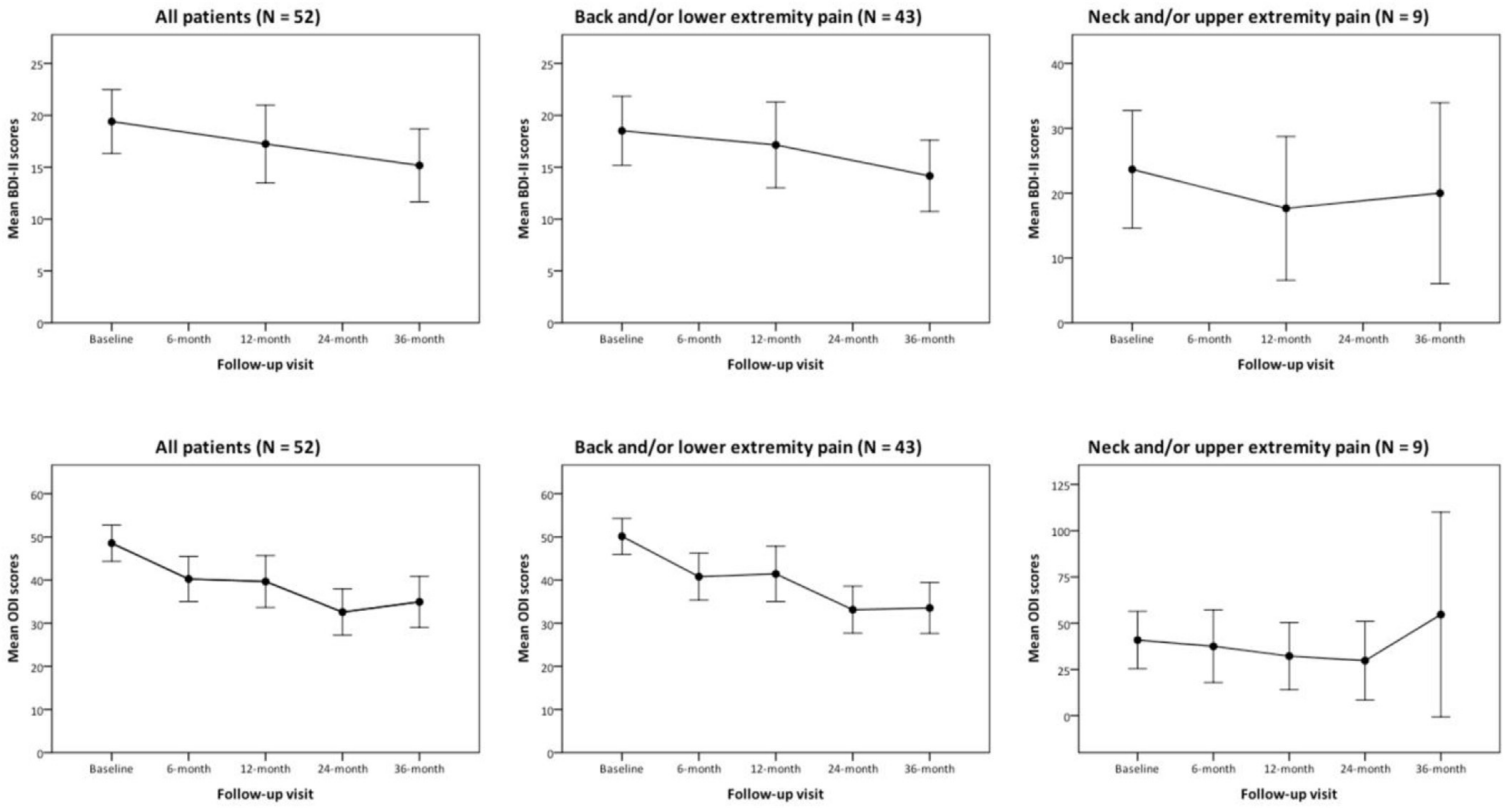
Evolution in mean BDI-II and ODI scores over time by indication for neurostimulation. BDI-II: Beck depression inventory – second edition; ODI: Oswestry disability index version 2.1a. Error bars represent 95% CI of the mean.

Shortly, after analyzing pain intensity and ODI scores change at 6, 12 and 24-month, along with BDI-II scores change at 12-month follow-up visit after implantation, we ob-served a significant decrease of pain intensity and ODI for both the whole cohort and the back and/or leg pain cohort at 6, 12 and 24-month visits (see Table 2, Table 3, Table 4 for details) (Fig. 3). Change in BDI-II from baseline to 12-month visit remained non-significant for those patients.

When considering the patients implanted because of neck and/or upper extremity pain, changes in pain intensity, ODI and BDI-II resulted non-significant at all three time points, with the exception of ODI at 12-month and arm pain at 24-month visits (P ​= ​0.038 and P ​= ​0.011 respectively).

### 3.4 Additional outcomes

Table 6 summarizes results regarding additional outcome measures at 6-, 12-, 24- and 36-month visits.

**Table 6:** Additional outcome measures at 6-, 12-, 24- and 36-month follow-up visits.

Variable^¶^	N	6 months	12 months	24 months	36 months	Statistic^a^	P-value^b^
All patients (N ​= ​52)							
Targeted paresthesia coverage (%; mean ​± ​SD)	19	65.26 ​± ​27.71	61.05 ​± ​25.20	71.32 ​± ​27.68	63.95 ​± ​27.31	0.818	0.490
**New areas [Yes; N (%)]**	45	18 (40)	16 (36)	12 (27)	6 (13)	9.333	0.025
**PGIC [N (%)]**						0.114	0.108
Very much worse		0 (0)	0 (0)	0 (0)	0 (0)		
Much worse		3 (6.1)	7 (14.3)	2 (3.8)	1 (2.0)		
Minimally worse		1 (2.0)	0 (0)	0 (0)	1 (2.0)		
No change		4 (8.2)	4 (8.2)	4 (7.7)	5 (9.8)		
Minimally improved		15 (30.6)	8 (16.3)	12 (23.1)	13 (25.5)		
Much improved		21 (42.9)	18 (36.7)	19 (36.5)	18 (35.3)		
Very much improved		5 (10.2)	12 (24.5)	15 (28.8)	13 (25.5)		
**CGIC [N (%)]**						0.112	0.112
Very much worse		0 (0)	1 (2.0)	0 (0)	0 (0)		
Much worse		2 (4.0)	5 (10.2)	2 (3.8)	2 (3.8)		
Minimally worse		1 (2.0)	2 (4.1)	1 (1.9)	1 (1.9)		
No change		5 (10.0)	1 (2.0)	5 (9.6)	3 (5.8)		
Minimally improved		18 (36.0)	15 (30.6)	11 (21.2)	16 (30.8)		
Much improved		17 (34.0)	14 (28.6)	21 (40.4)	16 (30.8)		
Very much improved		7 (14.0)	11 (22.4)	12 (23.1)	14 (26.9)		
**SST - Comfort of the stimulation [N (%)]**						0.040	0.576
Completely disagree		0 (0)	0 (0)	1 (2.0)	0 (0)		
Somewhat disagree		1 (2.0)	3 (6.1)	1 (2.0)	0 (0)		
Slightly disagree		4 (8.0)	1 (2.0)	2 (3.9)	3 (5.8)		
Neither agree nor disagree		7 (14.0)	5 (10.2)	3 (5.9)	2 (3.8)		
Slightly agree		3 (6.0)	2 (4.1)	8 (15.7)	8 (15.4)		
Somewhat agree		16 (32.0)	11 (22.4)	14 (27.5)	16 (30.8)		
Completely agree		19 (38.0)	27 (55.1)	22 (43.1)	23 (44.2)		
**SST - Amount of painful areas covered [N (%)]**						−0.055	0.436
Completely disagree		1 (2.0)	1 (2.1)	2 (3.9)	1 (1.9)		
Somewhat disagree		1 (2.0)	2 (4.3)	2 (3.9)	0 (0)		
Slightly disagree		2 (4.1)	0 (0)	3 (5.9)	5 (9.6)		
Neither agree nor disagree		4 (8.2)	4 (8.5)	2 (3.9)	3 (5.8)		
Slightly agree		7 (14.3)	6 (12.8)	12 (23.5)	12 (23.1)		
Somewhat agree		16 (32.7)	12 (25.5)	10 (19.6)	12 (23.1)		
Completely agree		18 (36.7)	22 (46.8)	20 (39.2)	19 (36.5)		
**SST - Overall therapy [N (%)]**						−0.013	0.860
Completely disagree		1 (2.0)	2 (4.2)	1 (2.0)	1 (1.9)		
Somewhat disagree		2 (4.1)	2 (4.2)	1 (2.0)	0 (0)		
Slightly disagree		1 (2.0)	1 (2.1)	4 (7.8)	3 (5.8)		
Neither agree nor disagree		5 (10.2)	4 (8.3)	2 (3.9)	4 (7.7)		
Slightly agree		6 (12.2)	4 (8.3)	13 (25.5)	12 (23.1)		
Somewhat agree		17 (34.7)	11 (22.9)	6 (11.8)	12 (23.1)		
Completely agree		17 (34.7)	24 (50.0)	24 (47.1)	20 (38.5)		

Abbreviations: PGIC: patient global impression of change; CGIC: clinician global impression of change; SST: satisfaction with stimulation treatment.

a F-statistic for repeated measures ANOVA, Q for Cochran's test and rho (ρ) for Spearman's rank-order test.

b Repeated measures ANOVA (within group comparisons), Cochran's Q test or Spearman's rank-order test.

Briefly, paresthesia coverage of targeted pain area did not show a significant change at any follow-up time point (P ​= ​0.490). Conversely, the development of new neurostimulation-amenable pain areas since implantation decreased along the study period (P ​= ​0.025). By means of pairwise comparison, significant differences were detected only between 6 and 36-month visits (P ​= ​0.028).

Both PGIC and CGIC values showed non-significant correlation with follow-up periods (Spearman's rho ​= ​0.114; P ​= ​0.108 and Spearman's rho ​= ​0.112; P ​= ​0.112 respectively).

Regarding SST dimensions, none showed a statistically significant correlation with follow-up time points. The comfort of the stimulation, the amount of painful areas covered and the overall satisfaction with the stimulation therapy dimensions of the SST did not show a significant correlation with follow-up visits (Spearman's rho ​= ​0.040; P ​= ​0.576; Spearman's rho ​= ​−0.055; P ​= ​0.436 and Speaman's rho ​= ​−0.013; P ​= ​0.860 respectively).

### 3.5 Adverse events and component device deficiencies

During follow-up a case of lead migration, a case of unsuccessful lead implant, a case of high impedance (all three required surgical revision), a case of lumbosciatic pain potentially related to the device (managed with medication) and a case of implant site infection (that required explant and additional system implanted) were retrieved. All five patients were able to reach the 36-month follow-up evaluation.

### 3.6 Linear regression

Opioid treatment and targeted pain intensity at baseline were identified by the linear regression model to be the independently associated with greater changes in targeted pain intensity from baseline to 36-month visit (B ​= ​3.293, P ​= ​0.026 and B ​= ​0.796, P ​= ​0.003 respectively). Statistically significant heteroskedasticity was discarded by means of the Breusch-Pagan test (P ​= ​0.146). Detailed information about the model and the rest of the independent variables is summarized in Table 7.

**Table 7:** Linear regression model for targeted pain intensity reduction (in NPRS points) from baseline to 36-month visit as dependent variable (F ​= ​3.943; P ​= ​0.001; R^2^ ​= ​0.688; Durbin-Watson ​= ​1.749).

Variable	B	t	95% CI for B	P-value	VIF
**Constant**	−3.145	−0.850	−10.769–4.479	0.404	-
**Opioids (N)**	3.293	2.375	0.437–6.149	0.026∗	1.621
**Diabetes (yes)**	2.857	1.407	−1.324–7.038	0.172	1.668
**Anticonvulsants (N)**	−2.184	−1.898	−4.553–0.186	0.069	1.454
**Gender (male)**	−2.182	−2.005	−4.423–0.060	0.056	1.331
**NSAIDs (N)**	1.149	0.732	−2.083–4.381	0.471	1.412
**Targeted pain at baseline (points)**	0.796	3.278	0.296–1.296	0.003∗	1.868
**Current smoker (yes)**	0.444	0.376	−1.989–2.876	0.710	1.251
**Previous spine surgeries (N)**	−0.109	−0.212	−1.161–0.944	0.833	1.514
**Time from pain to surgery (years)**	0.104	0.868	−0.142–0.350	0.393	2.341
**BMI (kg/m**^**2**^**)**	−0.079	−0.658	−0.325–0.168	0.517	1.590
**Age (years)**	0.031	0.677	−0.064–0.126	0.505	2.015
**BDI-II at baseline (score)**	−0.020	−0.377	−0.132–0.091	0.709	1.769
**ODI at baseline (score)**	−0.014	−0.336	−0.098–0.071	0.740	1.805
**SF-MPQ 2 ​at baseline (score)**	0.010	0.683	−0.021–0.041	0.501	1.474

Abbreviations: CI: confidence interval; VIF: variance inflation factor; NSAIDs: nonsteroidal anti-inflammatory drugs; BMI: body mass index; BDI-II: Beck depression inventory – second edition; SF-MPQ 2: short-form McGill pain questionnaire 2; ODI: Oswestry disability index version 2.1a, ∗ Statistically significant, P ​< ​0.05.

## 4 Discussion

The present study assesses the long-term effectiveness of SCS in routine clinical practice as an option for chronic back and neck pain. The results confirmed the effectiveness of SCS for as long as 36 months after implantation.

NPRS has been recommended by the Initiative on Methods, Measurement, and Pain Assessment in Clinical Trials (IMMPACT) consensus group to measure pain in clinical trials (Dworkin et al., 2005) and, more recently, the Critical Path's PRO Consortium also concluded that NPRS is the most optimal pain intensity measure for clinical trials in adults without cognitive impairment (Safikhani et al., 2018).

The IMMPACT consensus has also recommended considering 6 core outcome do-mains when assessing chronic pain therapies (Dworkin et al., 2005; Turk et al., 2003). In the present study we included an assessment tool for each and every core outcome domain, namely: (1) NPRS for pain, (2) ODI for physical functioning, (3) BDI-II for emotional functioning, (4) PGIC and SST for participant ratings of improvement and satisfaction with treatment, (5) follow-up clinical evaluation and interview for the capture of symptoms and adverse events and (6) detailed flow diagram of cohort generation for participant disposition.

The significant reduction in NPRS, BDI-II and ODI scores accounts for the improvement of patients in the physical, psychological and functional spheres. In fact, the mean difference for ODI values were over 15 points, a frequently used criterion of clinically significant improvement (Fairbank, 2014). Likewise, the BDI-II values improved >3 points on average, thus exceeding the limit usually considered to determine a significant clinical recovery in depression (National Institute for Health and Clinical Excellence, 2004). That improvement was observed both in the case of the whole cohort and in the case of the subgroup of patients implanted because of back and/or lower extremity pain. Only the subgroup of patients in whom the indication for SCS was either neck and/or upper extremity pain did not experience a significant improvement in pain intensity (with the exception of arm pain), BDI-II or ODI scores. The limited number of patients included in this subgroup may have contributed to those results. In our experience, cervical electrodes are more difficult to place and their indication often imply a more severe nervous lesion (e.g. root avulsion, traumatic spinal cord injury, etc.), so a selection bias could justify that those patients with more severe pathologies are selected for SCS, therefore resulting in a reduction of its effectiveness in this subgroup.

Regarding the intermediate follow-up visits at 6, 12 and 24 months, a significant, though more subtle, improvement in pain intensity and ODI values were also observed, especially when considering the 6- and 24-month visits. Interestingly, at 12 months, a mild stagnation in improvement was detected. It was more evident in the case of the BDI-II scores, that practically returned to levels similar to those observed at baseline. Classically, neuromodulation therapies have been thought to lose their effect over time. However, our observations suggest that studies with limited follow-up periods could come to identify temporary stagnation as a misleading tapering of the effects (Knotkova et al., 2021).

In recent years, several authors have tested different SCS systems in order to evaluate its efficacy and effectiveness for chronic pain (e.g. high frequency SCS, burst SCS, closed-loop SCS, etc) (Knotkova et al., 2021; Schu et al., 2014). Some of those studies use responder rates (e.g. number of patients with ≥50% pain reduction) as their main pain relief outcome measure (Brooker et al., 2021; Brinzeu et al., 2019).

Responder definitions are typically required within regulatory review because of their capacity to quantify treatment benefit and assess the meaningfulness of changes on the outcome measures. (Coon and Cook, 2018). However, dichotomising continuous variables can lead to several problems including loss of information, underestimation of outcome variability, concealing of non-linear relations and increased type I error (risk of a positive result being a false positive) (Altman and Royston, 2006; Austin and Brunner, 2004). Nevertheless, results of responder analyses are recommended for assessing clinical meaningfulness of group differences when independent-samples comparisons are made (e.g. double-arm clinical trials) (Dworkin et al., 2009). In the case of studies evaluating individual patient improvements, efforts to establish thresholds for clinically important changes in pain intensity rely on what patients themselves consider a meaningful improvement (Dworkin et al., 2009). The IMMPACT consensus statement on the clinical importance of treatment outcomes in chronic pain clinical trials (Dworkin et al., 2008) proposed an equivalence between NPRS score, percentual decreases in individuals’ pain intensity and type of improvement: 1 point or 10–20% decrease would correspond to a minimally important change; 2 points or ≥30% decrease would correspond to a moderately important change; 4 points or ≥50% decrease would correspond to a substantial change.

Therefore, in our study, mean targeted, leg and back pain decrease from baseline to 36-month visit would be classified as moderately-important-to-substantial changes.

Our results showed a steady percentage of paresthesia coverage of the targeted pain area. Minor, non-significant, fluctuations were observed along follow-up. That suggests that paresthesia coverage is not a parameter that needs to be progressively increasing in order to achieve progressively lower pain intensity results over time.

The results regarding PGIC and CGIC indicate that improvement brought up by SCS was patent for both the patient and the clinician since very early during treatment. Those high levels of improvement perception were maintained during long-term follow-up.

Regarding patient's SST, a great majority (more than 80%) of patients agreed, up to some extent, on a comfortable sensation of the stimulation along with a sufficient amount of painful areas' coverage and the overall satisfaction with therapy. The fact that those high levels of satisfaction were present since the first follow-up visit was in consonance with the significant improvement in pain intensity, BDI-II and ODI values already evident at that same time point. These observations accounts for the early and maintained effect of SCS therapy, that remained despite the above-mentioned fluctuations in pain intensity, BDI-II and ODI scores over time. In our opinion, that should support the maintenance of SCS treatments despite momentary stagnation or regression along follow-up.

Our study identified two factors independently associated with greater changes in targeted pain intensity by means of a linear regression model. The fact that one of these factors was the targeted pain at baseline would be explained by the greater margin for improvement that patients with higher pain intensities would have after implantation of the SCS system.

The logistic regression model also identified an increased reduction in pain intensity in those patients on opioids before SCS therapy. In line with the previous argument, it is more likely that patients with higher pain scores would require opioids in their treatment and, therefore, have greater margin for improvement when introducing SCS. Nevertheless, since opioids have a limited effect on neuropathic pain (Cahill et al., 2003) and neuropathic pain remains the main indication for SCS due to its unresponsiveness to other therapeutic modalities (Burchiel and Raslan, 2019), we could also hypothesize that the relation identified by the model could be due to an inadequate overprescription of opioids in our cohort. Those patients would experience an increased response to SCS since their medical treatment would have been poorly optimized initially. This idea is reinforced by the predominance of patients with previous spine surgeries and peripheral neuropathic pain diagnosis in our sample.

Taking all together, the results rendered by our regression model could correspond to those patients with the highest targeted pain scores at baseline that, consequently, required opioids as part of their treatment. Therefore, identifying those patients with higher pain intensities could help to offer SCS to those with the greatest potential benefit and, also, to potentially reduce the opioid prescription.

### 4.1 Study limitations

The limited number of patients, along with the fact that it represents the experience of a single center, prevents the generalization of the results obtained in the present study. Moreover, some of the results observed corresponded to a very small subgroup of patients (namely, the neck and/or upper extremity pain as indication for neurostimulation sub-group). We believe that, despite limited, our results could account for the consideration of including subgroup analysis regarding indication for neurostimulation in future studies. Future work on the subject could help us to elucidate whether there are technical, anatomical or physiological conditions that limit the effectiveness in the case of cervical electrodes.

The fact that we only used Boston Scientific's SCS systems contributes to the limited generalization of the assumptions extracted from the present article to other commercially available systems. Further studies assessing effectiveness of SCS in routine clinical practice should address this limitation.

The absence of a control group makes it impossible to distinguish the effect of SCS from the mere effect of time and spontaneous improvement on the outcome measures. Likewise, since this was a single-branch study, no comparison between different types of SCS could be performed. The net benefit provided by the Boston Scientific's systems in comparison with other systems available on the market could, therefore, not be elucidated.

## 5 Conclusions

The results obtained in the present study confirm the long-term effectiveness of SCS for the treatment of chronic pain, particularly leg and back pain. Changes in pain intensity from baseline to 36-month follow-up were moderately significant to substantial in magnitude. Likewise, the decrease in ODI and BDI-II scores were clinically significant after 24 and 36 months of follow-up respectively. Analyzing the evolution of the outcome measures throughout the follow-up period, we observed a stagnation of the improvement at 12 months. However, after that time, the patients continued to improve until the end of the 36-month follow-up. In our opinion, this plateau phase in the improvement curve should not discourage clinicians or patients when assessing the full potential effect that SCS treatment can provide in each case.

## Funding

This work was supported in part by grant PI2017/00361 from Instituto de Investigación Carlos III to MLGG, and grant B2017/BMD-3688 from the 10.13039/100012818Community of Madrid to MLGG.

## Institutional review board statement

The study was conducted according to the guidelines of the Declaration of Helsinki, and approved by the Ethics Committee of Hospital Universitario La Paz (protocol version 1.0, May 22nd, 2019, clinical trial A7007).

## Informed consent statement

Informed consent was obtained from all subjects involved in the study.

## Data availability statement

The data and study materials for this clinical study will be made available to other researchers in accordance with the Boston Scientific Data Sharing Policy (https://www.bostonscientific.com/).

## Declaration of competing interest

The authors declare the following financial interests/personal relationships which may be considered as potential competing interests: Maria Luisa Gandia Gonzalez reports financial support was provided by Carlos III Health Institute. Maria Luisa Gandia Gonzalez reports financial support was provided by 10.13039/100012818Community of Madrid. Maria Luisa Gandia Gonzalez reports a relationship with Boston Scientific Corporation that includes: consulting or advisory. Jose Francisco Paz Solis reports a relationship with Boston Scientific Corporation that includes: consulting or advisory.
